# Supplementary figures and images for: Maintenance of delay-period activity in working memory task is modulated by local network structure
Source: PLoS Comput Biol. 2024 Sep 3;20(9):e1012415. doi: 10.1371/journal.pcbi.1012415 (PMC11398668; doi:10.1371/journal.pcbi.1012415)

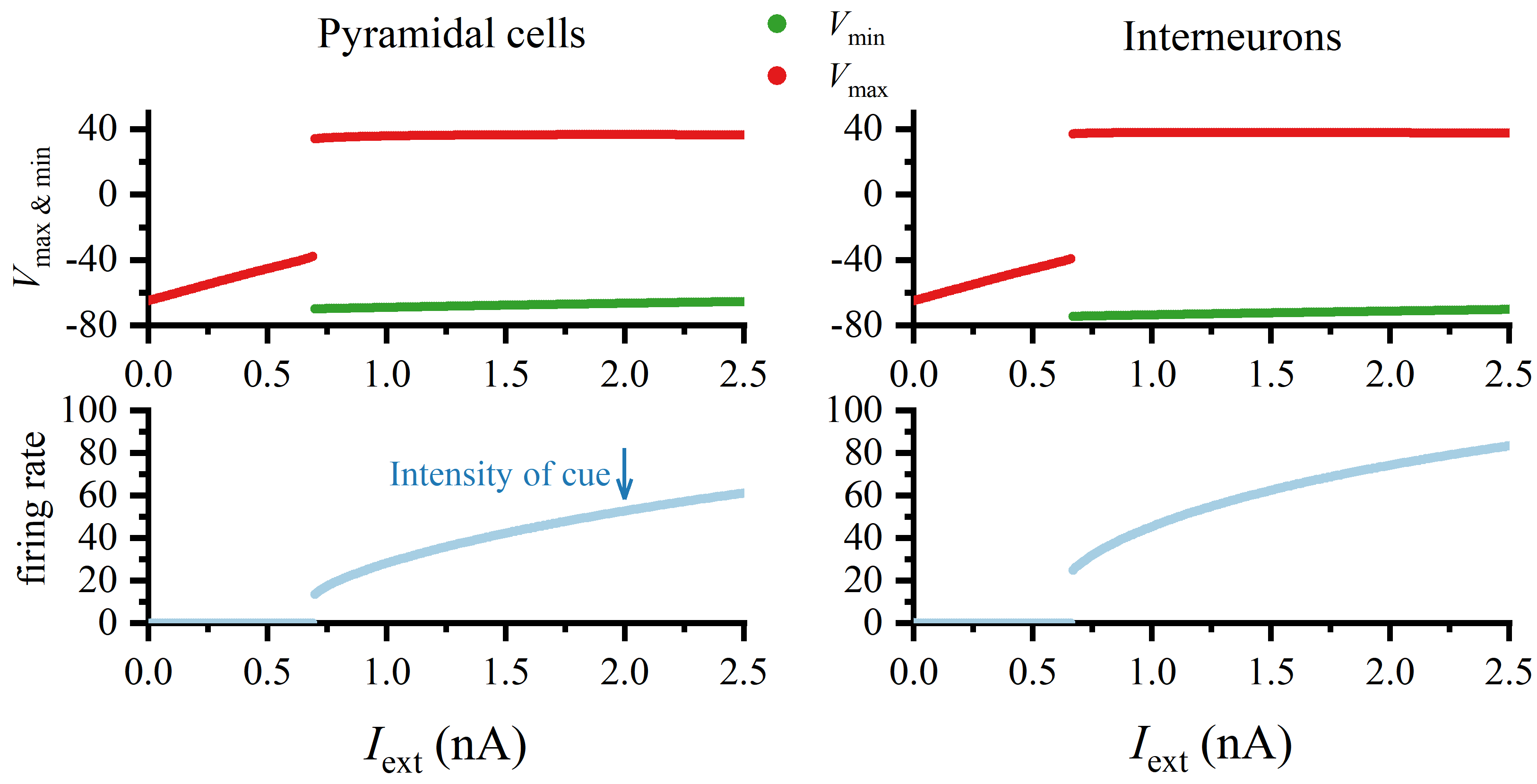

Supplement: S1 Fig — The firing thresholds of pyramidal cells and interneurons are both set at 0.7 nA. When a cue with an intensity of 2 nA is applied to pyramidal cells, neurons exhibit spiking state at a frequency of 52.7 Hz. (TIF) [file pcbi.1012415.s001.tif]

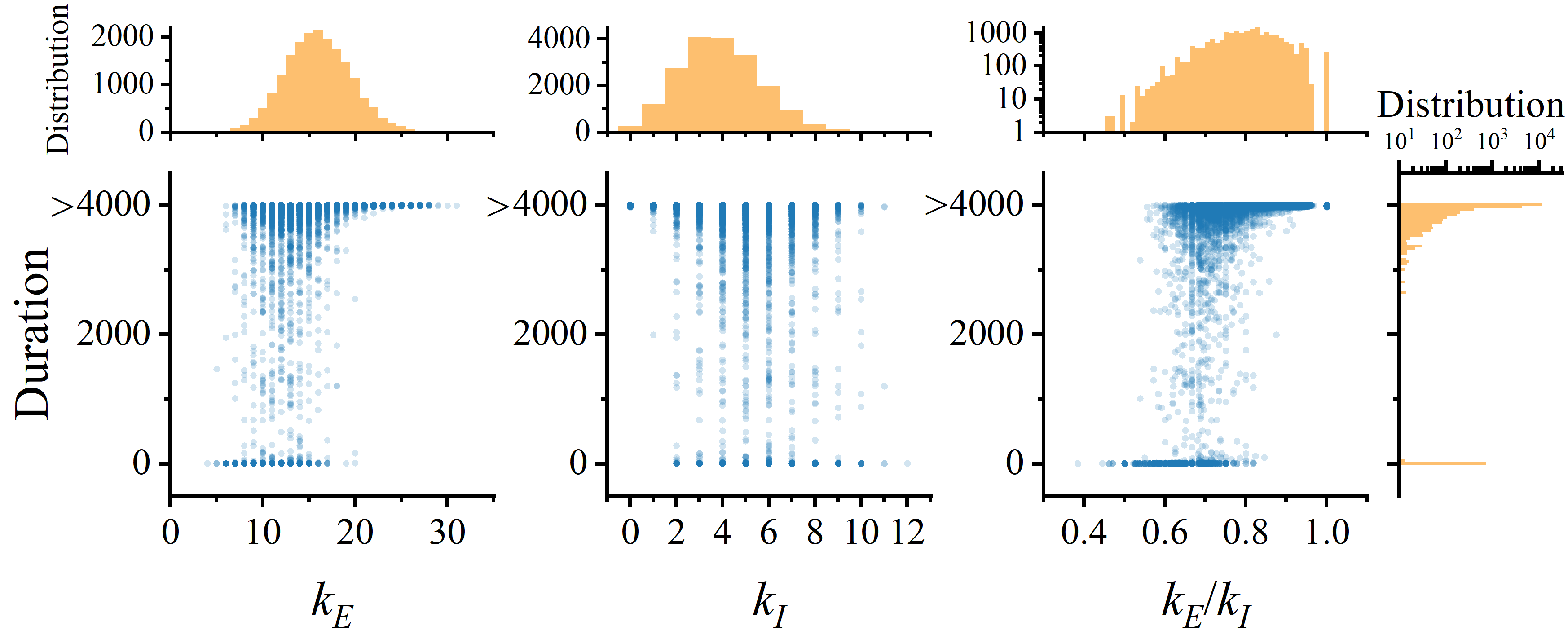

Supplement: S2 Fig — In 190 networks capable of sustaining electrical activity throughout the entire delay period, the duration of individual neuron’s electrical activity is correlated with the E/I ratio. The duration of neuron’s electrical activity exhibits a bimodal distribution (the right-side distribution subplot). Neurons with a high E/I ratio tend to sustain electrical activity, while those with a low E/I ratio terminate it rapidly. However, the generation of synaptic currents requires activation of presynaptic neurons. Thus, there exists a range of E/I ratio parameters where both silent and spiking neurons coexist. This suggests that beyond examining interactions between neurons pairwise, higher-order network interactions need to be considered. (TIF) [file pcbi.1012415.s002.tif]

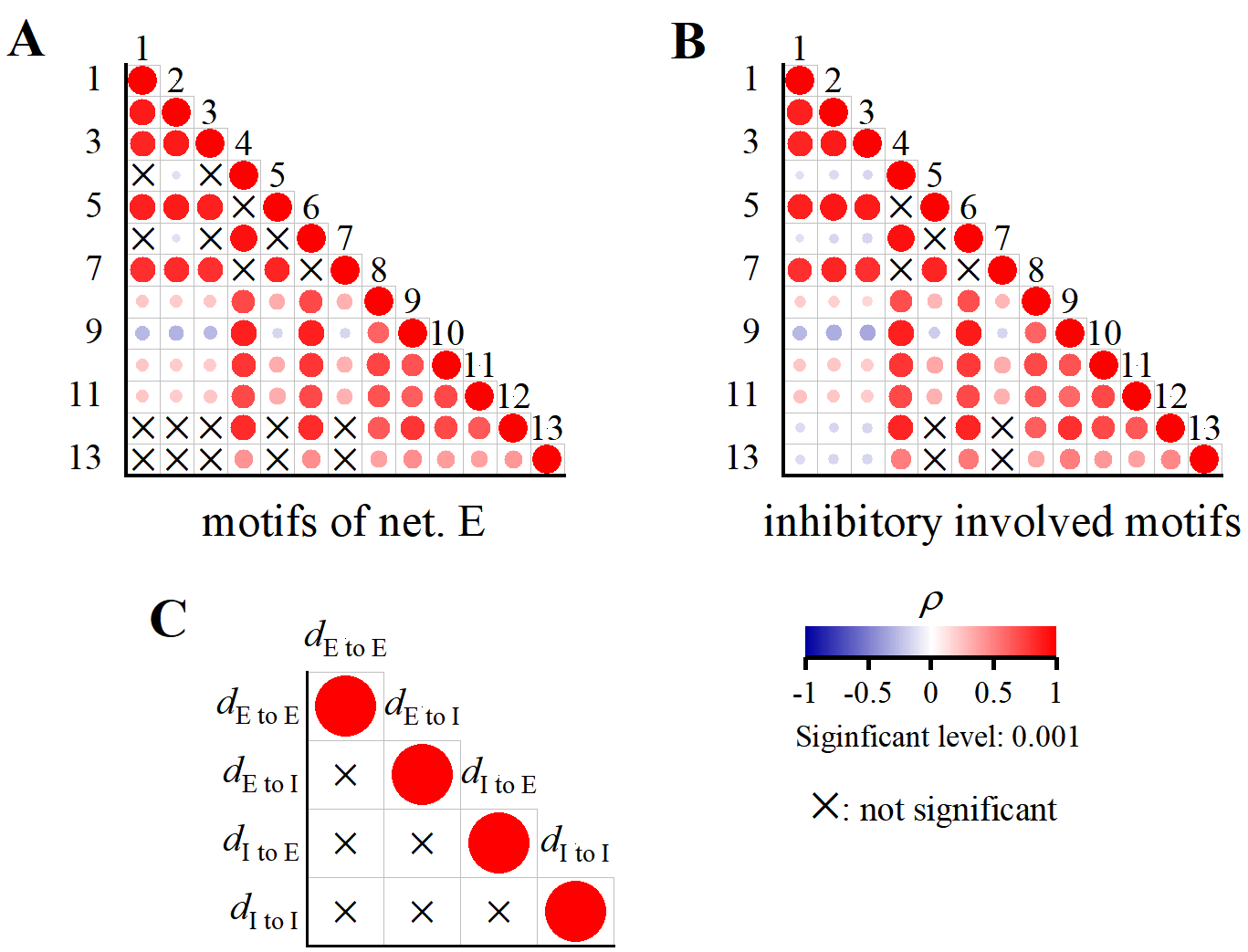

Supplement: S3 Fig — The size of the dots represents the correlation between variables. There is a high collinearity among the motifs in A) and B), while there is no collinearity among the in-degrees of neurons in C). (TIF) [file pcbi.1012415.s003.tif]

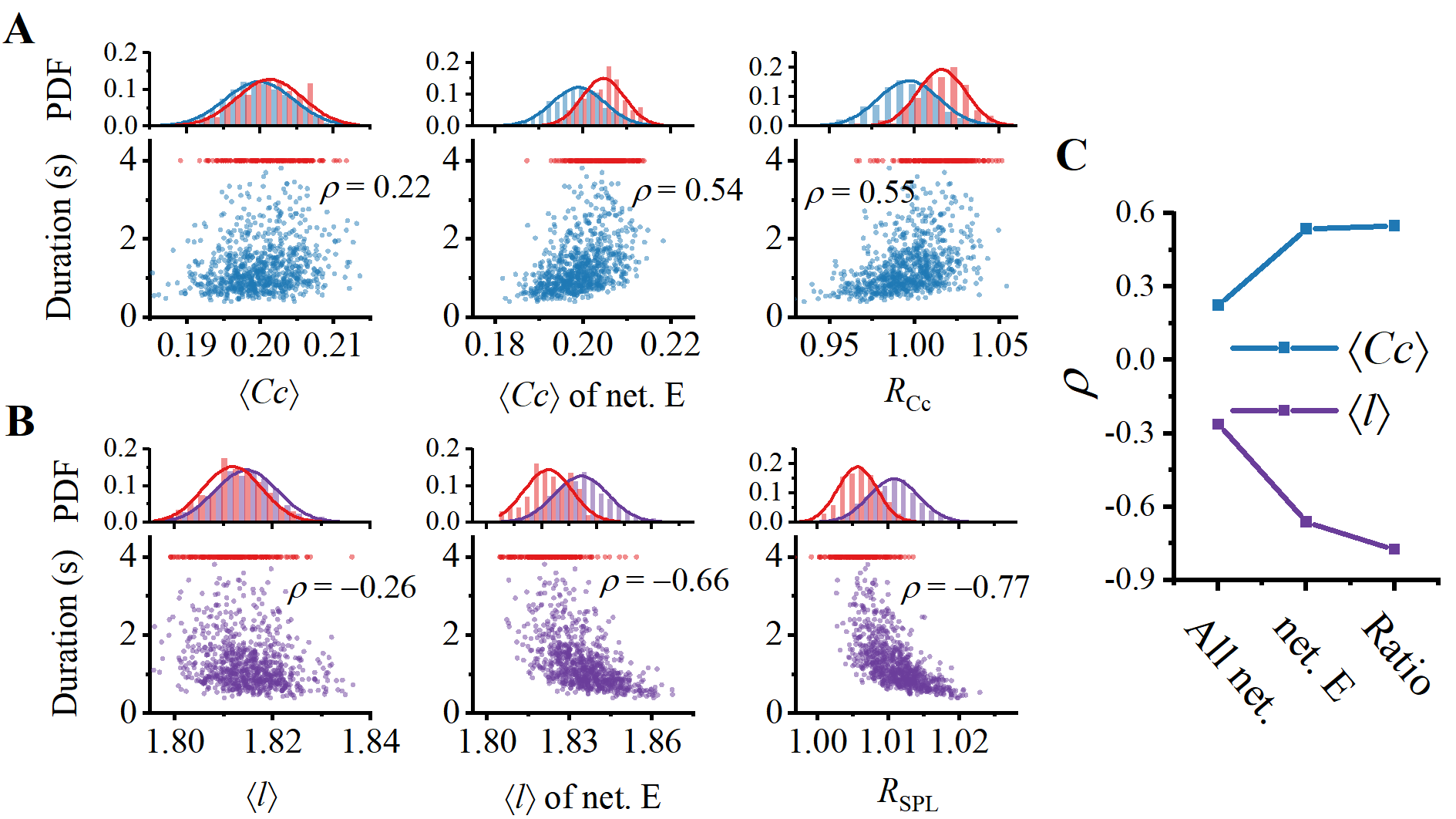

Supplement: S4 Fig — Dependence of duration of WM activity on A) clustering coefficient 〈Cc〉 and B) shortest path length 〈l〉 of network is explored. Topological properties of cortical networks are examined from three perspectives: entire network, excitatory subnetwork, and ratio R between the two. It is important to note that the choice of measured variables significantly impacts the correlation observed between these variables and duration of WM activity. C) Spearman correlation between small-worldness and duration of WM activity under three perspectives (p < 0.001 for all). Upon examining small-worldness in the entire network, we found that duration is almost uncorrelated with both Cc and SPL. Additionally, probability distributions of the two states exhibit a significant overlap (left panel). However, in excitatory subnetwork, results show a correlation, and the probability distributions of two states separate (middle panel). The relationship between the ratio of two networks (excitatory subnetwork and entire network) and the duration of WM activity reveals a stronger correlation (right panel), it can also be further validated in C), where duration is positively correlated with Cc and negatively correlated with SPL. (TIF) [file pcbi.1012415.s004.tif]

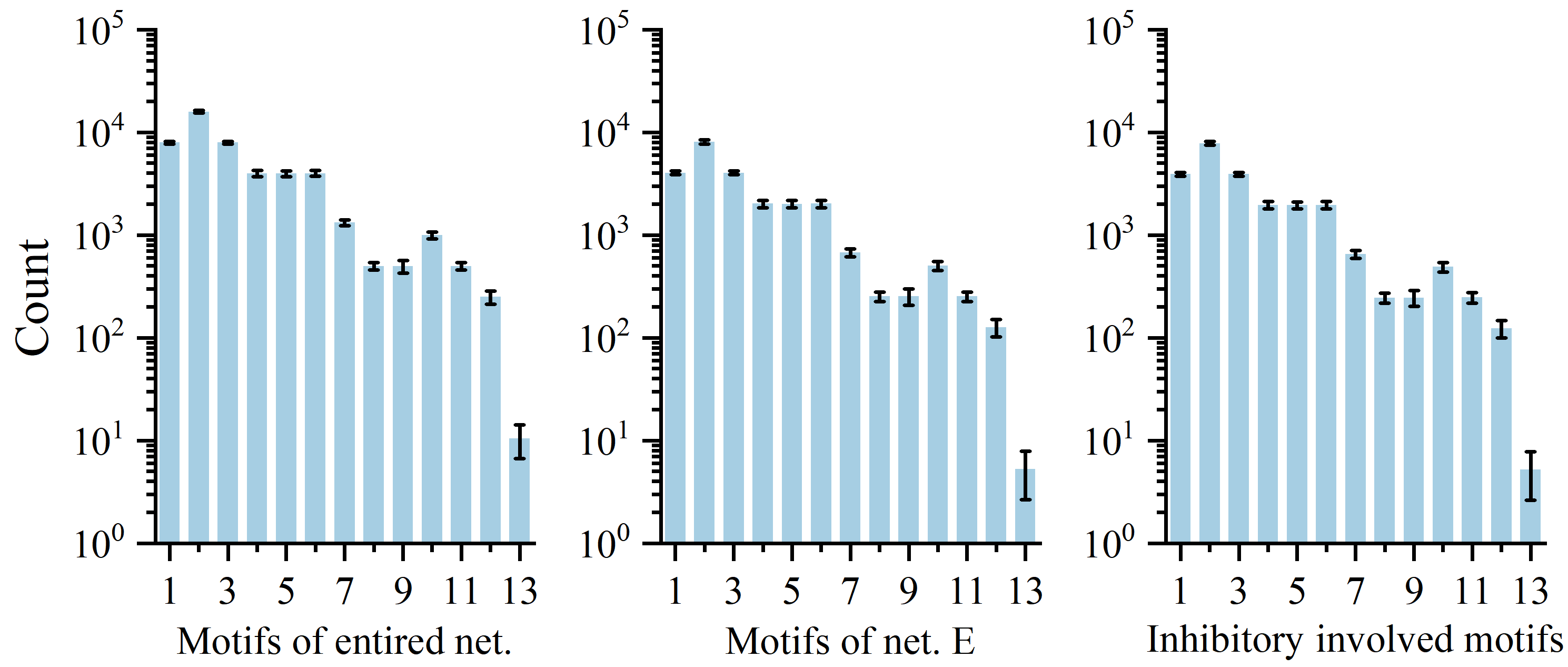

Supplement: S5 Fig — (TIF) [file pcbi.1012415.s005.tif]
