# Supplementary material for: Maintenance of delay-period activity in working memory task is modulated by local network structure
Source: PLoS Comput Biol. 2024 Sep 3;20(9):e1012415. doi: 10.1371/journal.pcbi.1012415 (PMC11398668; doi:10.1371/journal.pcbi.1012415)
Supplement: Attachment — Submitted filename: Response and Change.doc [file pcbi.1012415.s006.doc]

Your manuscript was reviewed by two reviewers. Both are positive about the work however they have raised some concerns which require a revision of the text to clarify some terminology, relate the model to biology of the brain networks and better align the work with the recent literature. Moreover, it is important that you share the code of the simulations. For more details please refer to the comments from the two reviewers. We look forward to reading your revision.

Reviewer's Responses to Questions

Comments to the Authors:

Please note here if the review is uploaded as an attachment.

Reviewer #1: Thank you! This was a nice read, related to my own work, so it was a pleasure. This paper should get published. Its a nice analysis how the (bi)stability of a biophysically detailed network is affected by by overall recurrent network connectivity, connection motifs, cycles, and receptor distributions. Some of these link to known cortical neuroscience, which makes this paper relevant to the field. The model description is reasonably clear, and the results presented in a straight-forward manner. However, I have some critical things to say that - in my mind -demand a few changes to the introduction and the discussion sections in particular, perhaps even the title.

Regarding the introduction

It is initially quite unclear what they mean by “synaptic gradients”, which could mean a lot of different things. Similarly, “aligning with neurotransmitter gradients within the WM network”, is quite unclear. These terms are not nearly as common as the authors perhaps assume, and deserve some concise two sentences of clarification to avoid reader confusion. For example, the later is not really a gradient of the neurotransmitter – like a chemical concentration gradient - , but much rather an experimentally observed gradient in the expression of specific transmitter receptors across the cortical hierarchy. The author summary, discussion and references eventually make this clear, but an introduction should not need an “introduction to the introduction”, but should clarify the scientific context of the study in unambiguous terms.

Reply: Thanks for your serious and responsible review of our manuscript. To clarify the meaning of synaptic gradient, we change the term “synaptic gradient” to “synaptic conductance gradient” and explain the neurotransmitter gradient:

“ The duration of delay-period activity depends on the synaptic conductance gradient, aligning with neurotransmitter gradients within WM network [39, 40]. The latter was observed experimentally in the transcript [39] and receptor densities [40] of the glutamate and GABA in macaque cortex. ” (Pag 5, lines 97-100 in the revised manuscript).

Topology also has a different meaning to researchers of the sensory cortical hierarchy, as topological connections preserve the nearest-neighbor relationships in the receptive fields across a sensory map across cortical areas. The authors instead use the term as synonymous with “structure” and based on graph-theoretical analysis of the recurrent connectivity within the local network, rather than across areas.

Reply: Thanks for your kind comments and suggestions. We have removed the term “topology” to avoid ambiguity, and the corresponding revision of the manuscript is highlighted in blue.

Regarding the Results:

The fact that activity becomes more persistent with small world topology, higher density of recurrent connectivity and its excitatory receptors AMPA and NMDA is qualitatively obvious. So is there a quantitative match with biology, or quantitative prediction in the conductance ranges they show? If so, this would enable further comparisons across models and electrophys data, but it seems that none of these are were biologically constrained, and the reader is left questioning the biological plausibility of the model details. What about the AMPA/NMDA charge ratio in biological cortical neurons(Myme et al. 2003; Rotaru et al. 2011), which would generally suggests much a much weaker NMDA conductance amplitude (NMDAR traces are of course much longer - 50x in this model - , delivering charge over time, rather than high amplitude like AMPA)? Is the model not at odds with the electro-physiological record here?

Reply: Thanks for your serious and responsible review of our manuscript. The conductance ranges we have discussed match experimental electrophysiological data. To clarify this, we have emphasized the comparison with electrophysiological data in the figure caption of Fig. 1:

“ Synaptic conductances were set to *g*_NMDA_ = 0.13 μS, *g*_AMPA_ = 0.11 μS, and *g*_GABA_ = 0.47 μS. The amplitude of the excitatory presynaptic currents of NMDA and AMPA were 19 pA and 71 pA, respectively, within the range of electrophysiological data [54, 55]. The NMDA/AMPA peak ratio is 26.6% consistent with what was observed experimentally [54]. ” (Pag 16, lines 318-322 in the revised manuscript).

The analysis of hub-neurons and triadic motives is graph-theoretically interesting and has merit in so far as it links directly to measurements of such quantities in the experimental record. The authors mention that record, and the model advances this discussion largely by providing a proof of concept for the role of these motifs in furthering recurrent activity. However, the authors do not attempt to match biology in any particular way here either, so this part remains a mere correlational analysis with little predictive or explanatory value beyond finding some biological analogies or correspondences.

Reply: Thanks for your kind comments and suggestions. We further discuss the advantages of the motif level at the scale of the computational function. We emphasize the perspective that functional clusters at the motif level are beneficial for improving the robustness of cortical computation.

“ Previous studies have suggested that some cognitive computational functions may occur at the motifs level [73-76]. Certain motifs can serve as inputs of long-term memory and thus play an important role in many cognitive processes. In addition, localized computation dominated by the motifs is robust to changes such as the loss of a single neuron, as other neurons can be recruited into the motifs to replace any lost neurons [76]. Thus, the computational scheme at the motifs level can provide robustness to the cortical computation. ” (Pag 30, lines 577-582 in the revised manuscript).

They model nicely predicts the different areas capacity for persistent activity, based on the expected recurrent connection density, yet given that the model network not clearly constrained otherwise, and the outcome mostly a threshold function on the spine count of basal dendrites in 2/3 layer pyramidal neurons of macaque, it is unclear wether the model is much more than a parameter tuning exercise.

Also: what about re-entrant effect across areas, i.e. the role of inter-area feedback, which may lead to reentrant dynamics? The methods mention that the model includes slightly abstracted interregional connections, but does not specify how exactly these connections are drawn, and what effects they have, which highly related models have found to be important for WM encoding and activity stability (Fiebig et al. 2018).

Reply: Thanks for your serious and responsible review of our manuscript. Spines are protruding structures on dendrites that make contact with the axons of other neurons, forming synaptic connections between neurons that transmit nerve signals. Therefore, we believe that spine data across areas most intuitively influences the synaptic connectivity probability of pyramidal neurons. To clarify this, we provided a detailed explanation of the reasons:

“ Layer 2/3 pyramidal cells in PFC have as many as 16 times more spines than in V1, as a result, the highly spinous cells in PFC may integrate many more inputs than cells in sensory areas such as V1, TE, and 7a [22, 23]. Spines are protruding structures on dendrites that make contact with the axons of other neurons, forming synaptic connections between neurons that transmit nerve signals. Therefore, the most direct effect of changes in spine count lies in input connectivity (or in-degree) of pyramidal cells. ” (Pags 30-31, lines 588-592 in the revised manuscript).

Moreover, as shown in Figure 8, for the occipital and temporal lobes and for much of the PFC, local features of the neural network (dominated by dendritic spines) have been sufficient to characterize delay-period sustained activity. As for the parietal and FEF (8L, 8M), our predictions were the opposite of what was observed experimentally. This demonstrates the need for cross-area re-entrant effects (global effects) in these brain regions to activate sustained activity during the delayed period. Therefore, our results emphasize the collaborative role of local and distributed (global) networks in brain neural networks.

We added a detailed scheme for cross-area connectivity:

“ In our model, it is approximated as a projection from pyramidal cells in sensory cortex to pyramidal cells in associative cortex. Feedback projections are more diffuse on their targets compared to forward-feed projections [46]. We hypothesize that feedback projections originate from pyramidal neurons in associative cortex and target all populations in sensory cortex. Therefore, as shown in Fig 1A, the projection from the association area to the sensory area (feedback) targets pyramidal cells and interneurons, whereas the projection from the sensory cortex to the association cortex (feedforward) targets only the pyramidal cells. Constrained by the number of basal dendritic spines in layer-3 of pyramidal cells, cross-area connections were similar to intra-area connections, with the reception probability set at 5% for pyramidal cells in the sensory area and 20% in the associated area. Reception probability of interneurons in the sensory area is fixed at 5%. Thus, we employ such connectivity rules to construct the adjacency matrix *M_A_^ext^* for interregional interactions. ” (Pag 8, lines 153-164 in the revised manuscript).

By normalizing biological spine counts the authors condition their connectivity merely on ratios of the quantitative experimental record, despite the fact that HH-neurons would allow for incorporation of a lot more fine-scale electrophysiological detail. Why use detailed biophysical neurons, if the goal is a mostly graph-theoretical analysis of node relationships?

Reply: Thanks for your kind comments and suggestions. We did not use the commonly used firing rate model because it has lost information about the local network structure. The leaky integrate-and-fire neuron model, which does not contain detailed electrophysiological details, was also not used by us. The reason is that LIF is a class-1 neuron model with continuous *f*-*I* curve, which exhibits slow firing activity in response to weak stimuli. Instead, we want to study the relationship between delay period activity and network structure. Baseline activity due to background noise is unnecessary. Therefore, we chose class-2 HH neurons with discontinuous f-I curves (shown in Fig. S1), whose baseline activity requires a higher background noise threshold. We highlight this in Method:

“ Our aim was to investigate the relation between the emergence and vanishing of delayed-period firing activity and the network structure in a WM task, where the Hodgkin-Huxley (HH) neuron model was employed to model the neurons. As a class-2 neuron model, it has a discontinuous *f*-*I* curve (shown in S1 Fig), whose baseline activity requires a higher background noise threshold [40, 41]. In contrast to class-1 neurons that fire slowly in response to weak stimuli (e.g., leaky integrate-and-fire neuron model, which has a continuous *f*-*I* curve), class-2 neurons easily eliminate baseline activity caused by background noise. We can focus more on the relationship between network structure and function. ” (Pag 6, lines 111-118 in the revised manuscript).

Regarding the Discussion and the Title, I see a paradigm-sized elephant in the room:

The idea that working memory function is primarily about persistent delay activity, and that such activity is synonymous with retention, is an increasingly questioned paradigm. Modern studies going far beyond the original ‘70s findings of persistent activity in macaque PFC during single-item delay maintenance, have since questioned how persistent any observed activity increases really are, if evidence for it are not mostly artifacts of large binning, intra- and inter-trial averaging, and reanalysis of old data has since shown that any increases in cell firing rates are generally much smaller (+3 Hz above baseline perhaps) than suggested by most recurrent network models, including this one (+30Hz or more for several seconds across an entire local network, which is unheard of in real recordings) (Shafi et al. 2007; Lundqvist et al. 2018).

Further, work of the last two decades has shown how these “persistent” signatures disappear with multi-item maintenance or distractors, without necessarily impacting eventual recall performance, when item-selective activity returns. This calls into question whether lasting activity is not primarily a signature of attention (or readout), rather than the memory engram itself. Ideas of “activity-silent” working memory(Stokes 2015), that may be revealed by unrelated strong stimuli(Rose et al. 2016), bursty-maintenance(Lundqvist et al. 2016), rely on synaptic memory (Mongillo et al. 2008; Lansner et al. 2023) and even local spatial computing in working memory networks(Lundqvist et al. 2023) have since gained traction in model work as well (Fiebig and Lansner 2017; Manohar et al. 2019).

A very critical reader may well conclude that the title of this paper is somewhat misleading. It is a careful graph-theoretical analysis of bistability in a biophysically detailed recurrent neural network. As such, it has merit, particularly where the model is actually constrained by neuroscience, but the link with working memory, as a term from cognitive science, is tenuous at best. Working memory implies much more than a single-item short term memory buffer, yet the authors do not even attempt to encode or maintain more than one item, apply distractors, dynamically update memory content, or use their “working memory” for any other task that might link tentatively to the executive functions implied by the term working memory. Given that the authors also uphold a now questionable paradigm about the centrality of persistent activity for WM maintenance, they should at least mention the ongoing controversy in their discussion rather than treat recurrent network (bi)stability as essentially synonymous with working memory.

Reply: Sincere thanks to the reviewer for reminding us. We scrutinized the entire manuscript, critically examining the term "working memory function" and removing inappropriate expressions. The existing controversies are discussed in the Conclusion section:

“ Our results highlight the dependence of delay-period activity on local network structure in WM tasks, which has been overlooked in previous studies. Furthermore, by adding rules affecting local structure, such as synaptic plasticity [10, 85], observing this structural and functional evolution can further understand the underlying mechanisms [86]. The concept that delayed-period activity maintains WM function is gradually being questioned. Delay-period activity in the PFC is not always essential for maintaining WM, and it can be re-established when attention is refocused on task-relevant content [87]. Therefore, WM does not seem to rely on delay-period activity, and is maintained in the form of "activity-silence". Lundqvist et al. demonstrated that WM exhibits discrete oscillatory dynamics and spiking, rather than sustained activity [88]. In addition, they propose a new concept of spatial computation in which beta and gamma interactions lead to delayed-period activity flowing spatially [89]. We do not emphasize that the maintenance of delay-period activity is equivalent to the WM function, but it intuitively depends on the local network structure. In addition, the WM function involves multiple processes. How the local network structure regulates specific WM functions, such as multi-task collaboration, task distractor, and dynamically update memory content, needs to be further explored in the future. ” (Pag 36-37, lines 674-689 in the revised manuscript).

Reproducibility

The most important aspects to rebuild this model from scratch are well laid out in the Methods, yet the Code-availability link is broken, it seems, which is a pity. I would have loved to take a look to find the answers to some of my questions myself.

Reply: Thanks for the reminder. We apologize for the code-share link being unavailable. We re-uploaded the code used to reproduce. Updated link to get the code: https://github.com/YuDong101/Structure_affects_delayed-period_activities

Thanks for your serious and responsible review of our manuscript and your valuable comments. We have revised the manuscript carefully, and the corresponding revisions of the manuscript are highlighted in blue. Your constructive suggestions have been very helpful to this manuscript and we hope that our revised manuscript will meet with your approval.

Reviewer #2: This is solid work, but poor presentation. I support the publication of this work, even as it is, but I recommend the authors restructure a bit the paper to increase impact. Instead of a long ling of things that impact WM duration, decide what are the main results and focus on that and move the rest to supplementary. Try as much as possible to merge or remove figures and have the strongest message possible on each figure.

Reply: Thanks for your kind comments and suggestions. We placed the studies on small-world organization (original Figs. 3B-D) into supplement. And placed the structure-induced bimodal distribution (original Fig. 3A) in Fig. 2, which makes Fig. 2 a clear representation of structure-induced bistability. In addition, we reorganized the study on rich hub neurons. The reorganized results (Fig 4) contain both single neuron scale and network scale results.

Minor:

1. introduce the model equations earlier in the text

Reply: Thanks for your kind comments and suggestions. We moved the method section before the result section.

2. Previous work focus on much simpler models, which seems good enough to study topological aspects of the connectivity. Justify (and highlight!) the choice for HH.

Reply: Thanks for your kind comments and suggestions. We did not use the commonly used firing rate model because it has lost information about the local network structure. The leaky integrate-and-fire neuron model was also not used by us. The reason is that LIF is a class-1 neuron model with continuous *f*-*I* curve, which exhibits slow firing activity in response to weak stimuli. Instead, we want to study the relationship between delay period activity and network structure. Baseline activity due to background noise is unnecessary. Therefore, we chose class-2 HH neurons with discontinuous f-I curves (shown in Fig. S1), whose baseline activity requires a higher background noise threshold. We highlight this in Method:

“ Our aim was to investigate the relation between the emergence and vanishing of delayed-period firing activity and the network structure in a WM task, where the Hodgkin-Huxley (HH) neuron model was employed to model the neurons. As a class-2 neuron model, it has a discontinuous *f*-*I* curve (shown in S1 Fig), whose baseline activity requires a higher background noise threshold [77, 78]. In contrast to class-1 neurons that fire slowly in response to weak stimuli (e.g., leaky integrate-and-fire neuron model, which has a continuous *f*-*I* curve), class-2 neurons easily eliminate baseline activity caused by background noise. We can focus more on the relationship between network structure and function. ” (Pag 6, lines 111-118 in the revised manuscript).

Thanks for your serious and responsible review of our manuscript and your valuable comments. We have revised the manuscript carefully, and the corresponding revisions of the manuscript are highlighted in blue. Your constructive suggestions have been very helpful to this manuscript and we hope that our revised manuscript will meet with your approval.
